# Eye movement patterns associated with colorectal adenoma detection: Post hoc analysis of randomized controlled trial

**DOI:** 10.1055/a-2549-1033

**Published:** 2025-04-04

**Authors:** Fumiaki Ishibashi, Kosuke Okusa, Mizuki Nagai, Kentaro Mochida, Eri Ozaki, Sho Suzuki

**Affiliations:** 1 38259 Department of Gastroenterology, International University of Health and Welfare Ichikawa Hospital, Ichikawa, Japan; 2 84021 Department of Data Science for Business Innovation, Chuo University Faculty of Science and Engineering Graduate School of Science and Engineering, Bunkyo-ku, Japan; 3 194321 Department of Gastroenterology, Shin Matsudo Central General Hospital, Matsudo, Japan

**Keywords:** Endoscopy Lower GI Tract, Polyps / adenomas / ..., Diagnosis and imaging (inc chromoendoscopy, NBI, iSCAN, FICE, CLE...), Quality and logistical aspects, Training

## Abstract

**Background and study aims:**

The adenoma detection rate is higher among endoscopists who spend more time observing screen edges during colonoscopies. Nonetheless, eye movement parameters related to lesion detection remain unknown. This study aimed to determine the specific eye movement parameters related to colorectal adenoma detection, including the gaze rate in a particular area and eye movement speed.

**Patients and methods:**

This study was a post hoc analysis of a randomized controlled trial investigating the effect of modifying eye movements of endoscopists on colorectal adenoma detection. Gaze rate at a specific area and eye movement speed were calculated based on endoscopist gaze coordinates in each examination. Time required for observation and treatment of polyps was excluded. The lower peripheral area was defined as the bottom row when the screen was divided into 6×6 sections. These parameters were compared between patients with and without adenomas.

**Results:**

Five physicians performed 158 colonoscopies. The adenoma detection group exhibited a lower peripheral gaze rate (13.7% vs. 9.5%, *P* = 0.004) and smaller average eye movement distance (29.9 pixels/30 ms vs. 33.3 pixels/30 ms, *P* = 0.022). Logistic regression analysis showed that a lower peripheral gaze rate > 13.0% and an average eye movement distance <30 pixels/30 ms were increased independent predictors of adenoma detection ( *P* = 0.024, odds ratio [OR] 2.53, 95% confidence interval [CI] 1.71-3.28; *P* = 0.045, OR 4.57, 95% CI 1.03-20.2), whereas age, sex, and withdrawal time were not.

**Conclusions:**

Lower peripheral gaze rate and slow eye movement are associated with colorectal adenoma detection.

## Introduction

Colonoscopy plays a pivotal role in detection of colorectal cancer (CRC), the second leading cause of cancer-related death 1 2 . The ability of colonoscopy to detect adenomas is a quality indicator in terms of reduction in interval CRC 3 4 . Therefore, improving measures such as the adenoma detection rate (ADR) and the number of adenomas per colonoscopy (APC) is crucial in clinical practice. Feedback to endoscopists about these parameters and colonoscopy withdrawal time is an efficient method of ensuring examination quality 5 6 7 ; however, ADR and APC greatly vary among endoscopists, even if withdrawal time is adequately achieved 5 .

Artificial intelligence has rapidly developed in the field of endoscopy, with various computer-aided detection (CADe) systems being used in daily clinical practice. Initial assessment of CADe in adenoma detection revealed that CADe effectively improved ADR or APC 6 . However, the effect size of CADe has been shown to depend on cohort characteristics. The most recent meta-analysis integrating real-world practice results concluded that CADe did not improve ADR and did not increase endoscopist burden 7 . This finding suggests that although CADe is an attractive tool, adenoma detection may be affected by endoscopists skills, which are not compensated for by CADe.

Eye-tracking technology is a promising tool for capturing the area where endoscopists gaze during an examination 8 . Analysis of endoscopist visual gaze pattern (VGP) during the withdrawal phase of colonoscopy has shown that the more frequently endoscopists gaze at the periphery of the screen, the higher their ADR 9 10 . In addition, VGP of expert and trainee endoscopists has been reported to differ significantly during observation 11 12 . A randomized controlled trial (RCT) successfully demonstrated that active modification of VGP could improve APC 13 and that peripheral gaze rate was higher in the intervention group than in the control group. Nevertheless, which VGP is related to adenoma detection remains unclear.

Determining the most influential VGP for adenoma detection would be useful in daily clinical practice. Furthermore, optimizing VGP management may further enhance ADR when used in conjunction with existing CADe systems. Therefore, the current study aimed to determine the VGP that contributed the most to adenoma detection via a detailed analysis of endoscopist gaze position coordinate information obtained in the RCT.

## Patients and methods

### Study design

This study was a post hoc analysis of an RCT conducted from July 2023 to December 2023. The International University of Health and Welfare Ethics Committee approved the study protocol on March 14, 2023 (approval number: 22-lm-040). Following the principles of the Declaration of Helsinki, written informed consent was obtained from all patients before their study enrollment.

### Participants

Patients aged 40 to 80 years who underwent colonoscopy for CRC screening at the International University of Health and Welfare Ichikawa Hospital were eligible for inclusion in the RCT. The exclusion criteria were as follows: a history of colorectal surgery, inflammatory bowel disease, hereditary or non-hereditary polyposis syndrome, known colorectal polyps or cancer, or inability to undergo total colonoscopy. This post hoc analysis included cases in which endoscopist eye position coordinate information and endoscopic videos were completely preserved without missing data.

### Procedures

Endoscopies were performed by five endoscopists with experience in performing > 3,000 colonoscopies over 5 years, who were certified as experts by the Japan Gastroenterological Endoscopy Society. The number of colonoscopies per endoscopist was limited to five per day, and all routine colonoscopies were performed in the afternoon. Endoscopy was performed using EC-760P and EC-760ZP colonoscopes with the ELUXEO 7000 system (Fujifilm Co., Tokyo, Japan). Use of chromoendoscopy or image-enhanced endoscopy, such as blue light imaging or linked color imaging, was not permitted during the scope withdrawal phase. To standardize recording of VGP, retroflexion observation and double pass observation of the right colon were not performed in any examination. Endoscopists recorded all detected polyps, except for hyperplastic polyps in the left colon. All identified adenomatous polyps and sessile serrated lesions (SSLs) were resected at the time of detection. These resected colorectal lesions were pathologically diagnosed. A nurse in the endoscopy room manually recorded observation time. Time required for the procedure, including polyp resection, was excluded from the observational period. Boston Bowel Preparation Scale (BBPS) score was used to evaluate the bowel cleansing level during examination 14 . The eye-tracking system was activated after reaching the cecum. The coordinate information of eye position and endoscopic video of withdrawal from the cecum to the rectum were recorded with perfect synchronization using a dedicated analysis system.

### Eye tracking

Eye movements of endoscopists were tracked and recorded using a dedicated eye-tracking technology-based system 13 . The system consisted of a screen-based eye tracker (Tobii Pro Spark; Tobii, Stockholm, Sweden) to obtain endoscopist VGP and a computer to integrate and analyze the data. Detailed specifications of this system can be found in the previous report 13 . The endoscopist viewpoint position was stored synchronously with the video in a green circle (diameter: 30 pixels). The coordinate information regarding endoscopist gaze position was recorded every 30 ms during the examination withdrawal phase. This included data collected during the screening of the colorectal lumen and during observation and treatment of lesions.

### Coordinate data processing

Representative video with eye-tracking and feedback (ETF) system. The green circle indicates endoscopist real-time viewpoint. The time at which the polyps were observed and treated was excluded from the analysis.Video 1

The video for each examination was reviewed to identify the time taken to find, observe, and treat polyps ( Video 1 ). Time required for observation and treatment of polyps was excluded from gaze position coordinate information by referring to the corresponding videos. After excluding time for observation and treatment of polyps, the generated coordinate data included only the time for observation of the colonic mucosa.

**Fig. 1:**
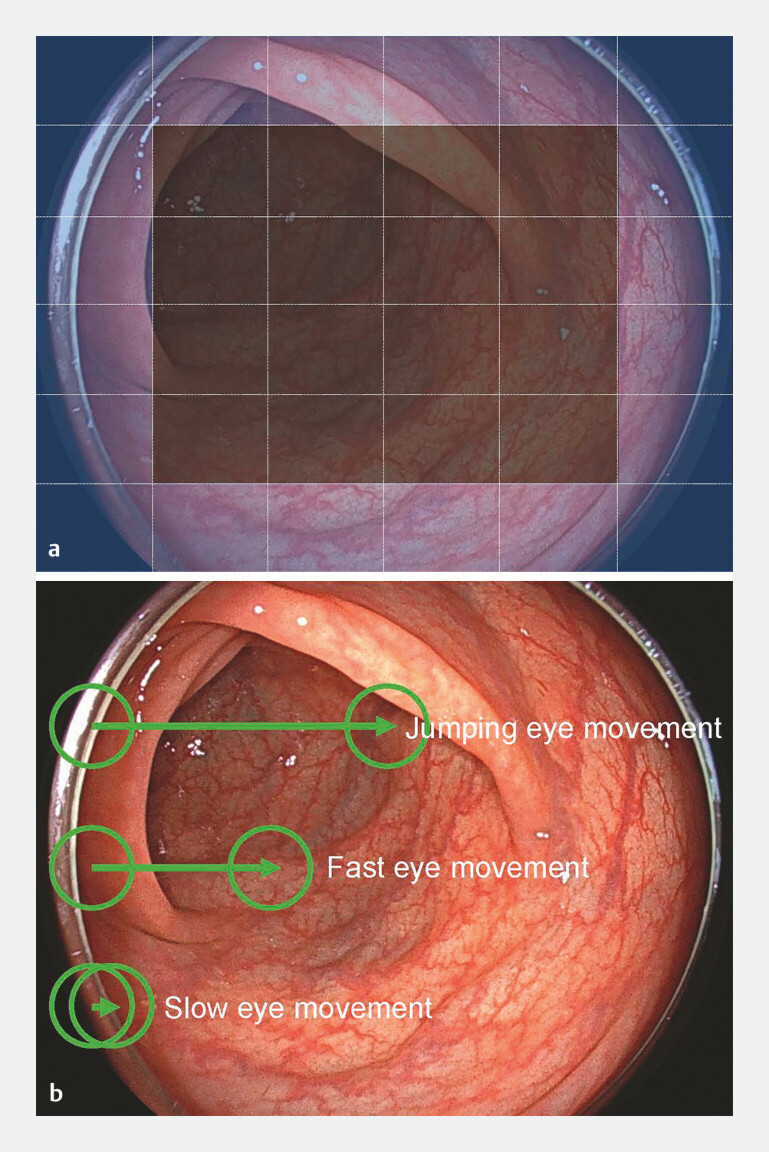
Parameter setting for eye movement in the analysis. **a** The endoscopy screen was divided into 36 (6×6) sections, of which 20 sections were defined as peripheral areas (blue sections). The top row of the peripheral area was defined as the upper peripheral area, whereas the bottom row of the peripheral area was defined as the lower peripheral area. **b** The green circle indicates endoscopist real-time viewpoint. The viewpoint shift where the Euclidean distance between two consecutive points was < 30 pixels/30 ms was defined as slow eye movement. The viewpoint shift, where it exceeded 300 pixels/30 ms, was defined as fast eye movement. The viewpoint shift exceeding 600 pixels/30 ms was defined as jumping eye movement.

First, the endoscopic image was divided into 36 6×6 sections; of these 36 sections, 20 were defined as peripheral areas ( Fig. 1 **a** ). The percentage of viewpoint locations within the peripheral area of all viewpoint locations was calculated as the peripheral gaze rate. The upper peripheral gaze rate was defined as the percentage of time at which the eye position was in the top row of the peripheral area. A lower peripheral gaze rate was defined as the percentage of time at which the eye position was in the bottom row of the peripheral area.

Next, the viewpoint-shift distance between two consecutive points was calculated by decomposing it into horizontal, vertical, and Euclidean distances. The average horizontal (Dx) and vertical (Dy) distances were calculated using the following equations:

$D_{x}=\frac{1}{n}\sum_{i=t}^{n}{(x}_{t+1}-x_{t})$

$D_{y}=\frac{1}{n}\sum_{i=t}^{n}{(y}_{t+1}-y_{t})$

The average Euclidean distance (D) of all viewpoint shifts for each inspection was determined using the following formula:

$\;D=\frac{1}{n}\sum_{i=t}^{n}\sqrt{({x_{t+1}-x_{t})}^{2}-({y_{t+1}-y_{t})}^{2}}$

where *x* indicates the x-axis coordinates, *y* indicates the y-axis coordinates, *n* indicates the number of eye movements, and *t* indicates time.

Slow eye movement was defined as a viewpoint shift in which the Euclidean distance between two consecutive points was < 30 pixels/30 ms; fast eye movement was defined as a viewpoint shift in which it exceeded 300 pixels/30 ms; and jumping eye movement was defined as a viewpoint shift in which it exceeded 600 pixels/30 ms ( Fig. 1 **b** ). Percentages of slow, fast, and jumping eye movements relative to the total number of movements were calculated.

### Data and statistical analyses

Colonoscopies included in the analysis were categorized into those in which adenomas were detected (adenoma-detected group) and those in which adenomas were not found (adenoma-not-detected group). First, patient background, examination data (insertion time, removal time, and degree of bowel cleansing), and gaze position coordinate information were compared between the two groups. Next, a logistic regression model was used to identify predictors, with adenoma detection as the objective variable and with patient background, examination data, and gaze position coordinate information as the explanatory variables. Owing to multicollinearity concerns, explanatory variables with a variance inflation factor (VIF) > 10 were excluded from multivariate analysis. Similarly, a logistic regression analysis was conducted to determine factors predicting detection of diminutive adenomas < 5 mm, small adenomas measuring 5 to 9 mm, and advanced adenomas. Advanced adenoma was defined as an adenoma that measured > 9 mm in size or had advanced histology or villous components.

Categorical variables were compared between the two groups using the chi-squared test or Fisher’s exact test. Continuous variables were compared using the Mann-Whitney U test or Student’s *t* -test. Results of the logistic regression analysis were expressed as odds ratios (OR) with 95% confidence intervals (95% CIs). Statistical significance was set at *P* < 0.05. All statistical analyses were performed using R version 4.0.4 (R Foundation for Statistical Computing, Vienna, Austria).

## Results

### Baseline characteristics

Coordinate data from 189 patients collected in the previous RCT were screened; 31 patients with missing data were excluded, resulting in a final dataset of 158 patients for analysis. Among them, 83 patients were classified as the adenoma-detected group, whereas 75 patients were assigned to the adenoma-not-detected group.

Characteristics of patients in both groups are summarized in Table 1 . The adenoma-detected group had significantly more males and longer withdrawal time than the adenoma-not-detected group (62.7% vs. 41.3%, *P* = 0.007; 523.5 ± 170.2 sec vs. 461.3 ± 101.2 sec, *P* = 0.007). No differences in other background characteristics, such as age and BBPS, were noted between the two groups. Lesion characteristics based on pathological results are presented in Supplementary Table 1. Of the 296 polyps identified in the video, 196 adenomas and 18 SSLs were included in the analysis.

**Table:** **Table 1** Baseline characteristics.

	Adenoma detected (n = 83)	Adenoma not detected (n = 75)	P value
Age	64.4 ± 10.7	60.8 ± 12.6	0.059
Sex (male)	52 (62.7)	31 (41.3)	0.007
BMI	22.9 ± 3.8	23.0 ± 3.7	0.818
Smoking habit (%)	43 (51.8)	36 (48.0)	0.633
Underlying diseases			
Diabetes mellitus (%)	7 (8.4)	14 (18.7)	0.059
Cardiovascular disease (%)	13 (15.7)	13 (17.3)	0.825
Constipation (%)	3 (3.6)	5 (6.7)	0.361
Thyroid disease (%)	5 (6.0)	5 (6.7)	0.834
Psychological disease (%)	10 (12.0)	16 (21.3)	0.099
Previous colonoscopy in 5 years	32 (38.6)	27 (36.0)	0.74
Insertion time (sec)	337.5 ± 197.2	335.9 ± 175.5	0.957
Withdrawal time (sec)	523.5 ± 170.2	461.3 ± 101.2	0.007
BBPS			
Total score	8.1 ± 1.2	8.1 ± 1.2	0.942
Right side	2.6 ± 0.5	2.6 ± 0.5	0.755
Transverse	2.7 ± 0.5	2.7 ± 0.5	0.813
Left side	2.8 ± 0.4	2.8 ± 0.4	0.634
BMI, body mass index; BBPS, Boston Bowel Preparation Scale. Previous colonoscopy was defined as colonoscopy performed within 5 years. Statistical significance was set at *P* < 0.05.			

### Predictors for adenoma detection

A detailed comparison of VGP between the adenoma-detected and adenoma-not-detected groups is shown in Table 2 . Similar to results of the previous RCT, lower peripheral gaze rate was significantly greater in the adenoma-detected group (13.7 ± 9.0% vs. 9.6 ± 8.2%, *P* = 0.004); however, peripheral and upper peripheral gaze rates did not differ between the two groups. Average eye movement Euclidean distance was significantly smaller in the adenoma-detected group, as were horizontal and vertical distances. As for eye movement speed, slow eye movement rate was substantially greater in the adenoma-detected group (79.8 ± 8.7% vs. 77.1 ± 7.7%, *P* = 0.045). Similarly, comparison for SSL detection showed that average eye movement Euclidean distance was significantly shorter and the slow eye movement rate was significantly higher in the SSL-detected group (26.6 ± 3.1 pixels/30 ms vs. 32.1 ± 9.6, *P* = 0.037; 82.6 ± 2.8% vs. 78.0 ± 8.5%, *P* = 0.049) (Supplementary Table 2).

**Table:** **Table 2** Comparison of visual gaze pattern.

	Adenoma detected (n = 83)	Adenoma not detected (n=75)	*P* value
Peripheral gaze rate (%)	17.9 ± 6.6	16.2 ± 6.2	0.082
Upper peripheral gaze rate (%)	2.8 ± 4.2	3.8 ± 5.9	0.229
Lower peripheral gaze rate (%)	13.7 ± 9.0	9.6 ± 8.2	0.004
Average distance			
Euclidean distance (pixel/30 ms)	29.9 ± 9.5	33.3 ± 9.0	0.022
Horizontal distance (pixel/30 ms)	18.5 ± 4.9	20.6 ± 4.8	0.008
Vertical distance (pixel/30 ms)	31.5 ± 8.2	35.5 ± 8.1	0.003
Slow movement rate (< 30 pixels) (%)	79.8 ± 8.7	77.1 ± 7.7	0.045
Fast movement rate (> 300 pixels) (%)	1.3 ± 0.6	1.5 ± 0.7	0.048
Jumping movement rate (> 600 pixels) (%)	0.2 ± 0.2	0.3 ± 0.3	0.072
*P* < 0.05 was considered statistically significant.			

A logistic regression model was applied, with age, sex, smoking history, withdrawal time, bowel cleansing level, gaze rate at a specific area, eye movement distance, and eye movement speed as explanatory variables and adenoma detection as the objective variable. Peripheral gaze rate and horizontal and vertical eye movement distances were excluded from the explanatory variables because their VIF exceeded 10. Logistic regression analysis revealed that lower peripheral gaze rate > 13.0% ( *P* = 0.024, OR 2.53, 95% CI 1.71–3.28), average eye movement distance of 30 pixels/30 ms ( *P* = 0.045, OR 4.57, 95% CI 1.03–20.27), and jumping eye movement ratio of 0.3% ( *P* = 0.014, OR 7.14, 95% CI 1.49–32.72) were independent predictors of adenoma detection; in contrast, patient background, such as age, sex, and withdrawal time, were not significant predictors ( Table 3 ). Next, we evaluated the impact of optimal VGP on detection of various types of polyps. In this analysis, we hypothesized that optimal VGP was a lower peripheral gaze rate ≥ 13% and an average eye movement distance < 30 pixels/30 ms. The results demonstrated that adenomas measuring 5 to 9 mm were significantly more frequently identified under the optimal VGP (Supplementary Table 3).

**Table:** **Table 3** Predictors for adenoma detection.

	*P* value	Odds ratio	95% CI
Age > 60 years	0.382	1.40	0.66–2.97
Male sex	0.07	2.05	0.94–4.48
Smoking habit	0.208	1.67	0.75–3.74
Withdrawal time > 420 sec	0.393	0.71	0.33–1.54
BBPS > 6	0.324	1.74	0.58–5.26
Upper peripheral gaze rate < 3.0%	0.663	0.82	0.35–1.96
Lower peripheral gaze rate > 13.0%	0.024	2.53	1.71–3.28
Average distance < 30 pixels	0.045	4.57	1.03–20.27
Slow movement rate > 79.0%	0.699	1.27	0.38–4.29
Fast movement rate < 1.4%	0.134	2.93	0.72–11.93
Jumping movement rate < 0.3%	0.014	7.14	1.49–32.72
BBPS, Boston Bowel Preparation Scale; CI, confidence interval. *P* < 0.05 was considered statistically significant.			

VGPs according to fast and slow withdrawal times were further analyzed for all participants. The results showed no significant differences in VGP parameters between the fast and slow withdrawal time groups ( Table 4 ).

**Table:** **Table 4** Comparison of visual gaze pattern by withdrawal time.

	WT ≥ 480	WT < 480	*P* value
Peripheral gaze rate	17.1 ± 6.3	17.0 ± 6.6	0.997
Upper peripheral gaze rate	4.4 ± 6.6	3.3 ± 4.4	0.071
Lower peripheral gaze rate	10.3 ± 7.2	13.0 ± 10.0	0.061
Average distance			
Euclidean distance (pixel/30 ms)	32.9 ± 11.7	30.4 ± 6.4	0.884
Horizontal distance (pixel/30 ms)	19.9 ± 6.0	19.2 ± 3.8	0.724
Vertical distance (pixel/30 ms)	34.4 ± 10.3	32.6 ± 6.0	0.167
Slow movement rate (< 30 pixels) (%)	77.1 ± 10.2	79.7 ± 5.7	0.053
Fast movement rate (> 300 pixels) (%)	1.5 ± 0.8	1.3 ± 0.5	0.292
Jumping movement rate (> 600 pixels) (%)	0.3 ± 0.3	0.3 ± 0.1	0.208
WT, withdrawal time. *P* < 0.05 was considered statistically significant.			

### Predictors of adenoma detection according to polyp characteristics

The same logistic regression model was applied, dividing the objective variables into diminutive adenoma < 5 mm, small adenoma measuring 5 to 9 mm, and advanced adenoma with 10 mm or advanced histology ( Table 5 ). A lower peripheral gaze rate was an independent predictor for detection of all adenoma types (OR 2.64, 95% CI 1.58–4.63; OR 2.90, 95% CI 1.86–4.18; OR 2.24, 95% CI 1.50–3.06). A jumping eye movement ratio < 0.3% was an independent predictor for detection of diminutive adenomas and small adenomas (OR 15.63, 95% CI 2.26–106.38; OR 5.70, 95% CI 1.17–27.72). An average eye movement distance < 30 pixels/30 ms was an independent predictor for detection of advanced adenomas (OR 6.80, 95% CI 1.03–44.92).

**Table:** **Table 5** Predictors of adenoma detection according to polyp characteristics.

Odds ratio (95% CI)	Adenoma < 5 mm	Adenoma 6–9 mm	Advanced adenoma
Age > 60 years	2.36 (0.78–7.19)	1.68 (0.77–3.70)	1.62 (0.63–4.15)
Male sex	0.63 (0.21–1.85)	2.37 (1.03–5.43)	2.46 (0.91–6.61)
Smoking habit	2.28 (0.75–6.98)	1.33 (0.58–3.08)	1.60 (0.61–4.18)
Withdrawal time > 420 sec	1.15 (0.40–3.28)	0.91 (0.41–2.01)	0.31 (0.12–0.79)
BBPS > 6	1.05 (0.24–4.63)	7.22 (1.67–31.30)	1.84 (0.43–7.89)
Upper peripheral gaze rate < 3.0%	0.98 (0.30–3.15)	1.05 (0.44–2.54)	1.18 (0.42–3.30)
Lower peripheral gaze rate > 13.0%	2.64 (1.58–4.63)	2.90 (1.86–4.18)	2.24 (1.50–3.06)
Average distance < 30 pixels	7.77 (0.70–85.67)	2.30 (0.49–10.89)	6.80 (1.03–44.92)
Slow movement rate > 79.0%	2.12 (0.35–12.94)	2.19 (0.61–7.82)	0.66 (0.14–3.16)
Fast movement rate <1.4%	0.85 (0.11–6.33)	3.41 (0.75–15.51)	0.90 (0.17–4.81)
Jumping movement rate < 0.3%	15.63 (2.26–106.38)	5.70 (1.17–27.72)	2.75 (0.60–12.55)
BBPS, Boston Bowel Preparation Scale; CI, confidence interval. Values are expressed as odds ratios (95% confidence intervals). *P* < 0.05 was considered statistically significant.			

## Discussion

Several factors influence the ability of an endoscopist to detect lesions during colonoscopy. Poor bowel cleansing worsens adenoma detection owing to decreased lesion visibility 15 16 . Fast withdrawal time is an indirect quality indicator because it is a sign that the mucosa is not well observed 17 18 19 . However, even if withdrawal time is increased beyond a certain threshold, improvement in ADR is small 18 19 . Similarly, ADRs vary widely among endoscopists, even for the same degree of bowel cleansing and withdrawal time, and are not related to endoscopist years of experience 5 . Thus, degree of bowel cleansing and withdrawal time are insufficient indicators for evaluating observation accuracy.

The specific VGP of endoscopists, which could serve as an indicator of observation accuracy, was explored. Eye-tracking technology allows us to determine exactly what part of the screen an endoscopist is looking at. Previous studies using eye-tracking technology for endoscopy have investigated whether the percentage of gazing at a particular area of the screen is associated with quality indicators, such as the ADR, by analyzing which areas of the screen were examined. The results showed that physicians who spent more time looking at the edges of the screen than at its center had a higher ADR 11 and endoscopists who looked at the bottom U region of the screen had a higher ADR 9 . Experts spend more time looking at the edges of the screen than non-experts 10 12 . Our previous RCT also demonstrated that guiding the gaze to screen edges improved APC and ADR 13 . Furthermore, in this RCT, no differences were observed in degree of bowel cleansing or withdrawal time between the control and intervention groups. These results indicate that percentage of gazing at screen edges is a candidate accuracy index for observation during colonoscopy. However, percentage of gaze at a specific area of the screen is an index that sums up the VGP throughout the withdrawal phase and does not express the moment of adenoma detection. In other words, previous studies have been unable to analyze VGP over time, making it difficult to analyze VGP other than percentage of gazing at a specific screen area.

The real-time VGP analysis system we developed provides time-stamped coordinate information, enabling more detailed VGP analysis than was possible in previous studies. In this study, percentage of gaze at a specific area of the screen and eye movement distance were broken down into units of time, and eye movement speed was calculated for all examinations. A simple comparison of the adenoma-detected and adenoma-not-detected groups showed a significant difference in percentage of participants gazing at the lower screen edge, as previously reported. Logistic regression analysis using detailed eye movement measures revealed that a higher percentage of gazing at the lower screen edge, shorter eye movement distance, and fewer jumping eye movements predicted adenoma detection. Importantly, multivariate regression analysis did not identify bowel preparation or withdrawal time as significant variables. In addition, there were no significant differences in VGP parameters between the fast and slow withdrawal time groups. These findings suggest that VGP during the scope withdrawal phase may represent a distinct quality parameter independent of withdrawal time. VGP with a short eye movement distance and a low frequency of jumping eye movement means a VGP with slow eye movement. Our results indicate that observation of slow eye movement < 30 pixels/30 ms is essential for adenoma detection.

In this study, known predictors of adenoma detection, such as male sex, older age, smoking habits, and withdrawal time, were not identified as independent predictors. Lack of association with age may be attributed to the relatively older mean age of the included patients, making it difficult to detect a significant difference. In addition, the high overall percentage of patients with smoking habits may have obscured its impact on adenoma detection. Furthermore, because almost all examinations in this study achieved a withdrawal time of 6 minutes or longer, withdrawal time may have been less of a risk factor because of the high quality of the examinations.

For quality control in screening colonoscopy, the SSL detection rate has been proposed as a separate target from ADR 20 . Results of our VGP analysis indicate that specific gaze patterns, including slow eye movement, are also associated with detection of difficult-to-identify flat lesions, such as SSLs. By optimizing VGP, it may be possible to achieve a high ADR and SSL detection rate, ensuring high-quality colonoscopies.

Time from the appearance of a polyp on the screen to endoscopist recognition of the polyp was reported to be 2.97 seconds on average, based on results of eye-tracking analysis using a recorded video 21 . Lesion identification performance may be reduced if lesions appear at > 2.97 seconds after the lesion appears. The screen used in our study was set to a horizontal diameter of 1230 pixels and a vertical diameter of 1080 pixels. Therefore, it takes 1.23 seconds to scan the entire horizontal direction and 1.08 seconds to scan the entire vertical direction at an eye movement speed of 30 pixels/30 ms. Scanning at an eye-movement speed of approximately 30 pixels/30 ms, even while withdrawing the endoscope, provides a high probability of identifying a lesion within 2.97 seconds after it appears on the screen. Thus, even if endoscopists were to move their gazes slowly, they would be unlikely to miss a lesion.

Although time from input to perception of visual information is not precisely known, it is estimated to be 100 to 400 ms, based on a comparison of behavioral reaction times and the time course of evoked magnetoencephalographic responses 22 . This means that even if a lesion is present, if the viewpoint does not coincide with the lesion for at least 100 ms, the lesion may not be recognized as a lesion. A detailed analysis by polyp size revealed that factor predicting detection of diminutive and small adenomas were the rate of jumping eye movement (< 0.3%). Endoscopists cannot recognize such small lesions if the viewpoint is not aligned with the lesion for more than 100 ms because the gaze passes over the lesion during the jumping eye movement.

Advanced adenoma detection rates (AADRs) vary widely among endoscopists and are independent of ADR 23 . Significantly, a low AADR is associated with interval CRC, even if sufficient ADR is achieved 24 . CADe, which has recently become widely used in screening colonoscopy, has been shown to aid in adenoma detection without increasing endoscopist burden. However, it has been suggested that CADe may not contribute to advanced adenoma detection 25 . The results of this study indicate that slow eye movement < 30 pixels/30 ms is essential for detecting not only all adenomas but also advanced adenomas. It would be a simple intervention if the AADR could be improved by making endoscopists aware of slowly moving their gaze for observation. Implementing VGP interventions while using CADe may enhance multiple quality indicators, including ADR, AADR, and SSL detection rates.

This study has some limitations. First, the data may have been biased because the previous study from which the data were derived was an RCT conducted by only five physicians at a single institution. Second, time from detection to resection of colorectal polyps, but not time spent aspirating the residue, was excluded from analysis. However, given that no difference in degree of bowel preparation was noted between the adenoma-detected and adenoma-not-detected groups, time taken to aspirate the residue was considered similar. This has little impact on results of the analysis. Third, because the endoscopist performing the examination was predetermined by the day of the week, potential bias could have been introduced if differences in age groups, sex, or other factors varied by examination day.

## Conclusions

In conclusion, a VGP analysis based on eye-tracking technology showed that a lower peripheral gaze rate and slow eye movement during the withdrawal phase of colonoscopy were associated with detection of adenomas, including advanced adenomas.

## Supplementary Material

Supplementary Material Zusatzmaterial
