# Supplementary material for: Eye movement patterns associated with colorectal adenoma detection: Post hoc analysis of randomized controlled trial
Source: Endosc Int Open. 2025 Apr 4;13:a25491033. doi: 10.1055/a-2549-1033 (PMC11996023; doi:10.1055/a-2549-1033)
Supplement: Supplementary file 2 — Supplementary Material [file 10-1055-a-2549-1033_25502693.pdf]

Supplementary Table 1 Lesion characteristics of identified polyps.

	Number of polyps (%)
All polyp	296
Right side	173 (58.4)
Left side	123 (41.6)
Adenoma	196
Right side	115 (58.7)
Left side	81 (41.3)
< 5 mm	31 (15.8)
5-9 mm	106 (54.1)
> 9mm	61 (31.1)
Flat type	85 (43.4)
Sessile type	111 (56.6)
Sessile serrated lesion	18
Right side	15 (83.3)
Left side	3 (16.7)
< 5 mm	2 (11.1)
5-9 mm	12 (66.7)
> 9mm	4 (2.2)
Flat type	15 (83.3)
Sessile type	3 (16.7)

Endosc Int Open | DOI 10.1055/a-2549-1033 | © The Author(s).

Supplementary Table 2 Comparison of visual gaze pattern by SSL detection.

	SSL detected (n= 14)	SSL not detected (n= 140)	*P* value
Peripheral gaze rate	18.8 ± 6.0	16.9 ± 6.5	0.283
Upper peripheral gaze rate	1.5 ± 2.1	3.5 ± 5.3	0.174
Lower peripheral gaze rate	15.5 ± 9.8	11.4 ± 8.7	0.095
Average distance			
Euclidean distance (pixel/30 ms)	26.6 ± 3.1	32.1 ± 9.6	0.037
Horizontal distance (pixel/30 ms)	16.9 ± 2.0	19.8 ± 5.0	0.031
Vertical distance (pixel/30 ms)	28.7 ± 3.2	33.9 ± 8.6	0.026
Slow movement rate (< 30 pixels) (%)	82.6 ± 2.8	78.0 ± 8.5	0.049
Fast movement rate (> 300 pixels) (%)	1.1 ± 0.2	1.4 ± 0.7	0.064
Jumping movement rate (> 600 pixels) (%)	0.2 ± 0.1	0.3 ± 0.2	0.067

SSL, sessile serrated lesion

P < 0.05 was considered statistically significant.

Supplementary Table 3 Number of polyps detected under the specific visual gaze pattern.

	**Lower peripheral gaze rate < 13% + eye movement distance ≥ 30 pixel/30 ms (n = 17)**	**Lower peripheral gaze rate ≥ 13% + eye movement distance < 30 pixel/30 ms (n = 29)**	***P* value**
Number of polyps	2.2 ± 1.6	3.6 ± 3.1	0.132
Right side	1.2 ± 1.4	2.1 ± 1.9	0.142
Left side	1.1 ± 1.0	1.6 ± 1.8	0.304
Number of adenomas	1.7 ± 1.0	2.4 ± 1.3	0.075
Right side	0.9 ± 0.8	1.4 ± 1.2	0.136
Left side	0.8 ± 0.8	1.0 ± 1.2	0.585
< 5 mm	0.3 ± 0.5	0.4 ± 0.8	0.57
5-9 mm	0.9 ± 0.7	1.5 ± 1.2	0.046
> 9 mm	0.5 ± 0.9	0.4 ± 0.6	0.717
Number of SSLs	0.1 ± 0.3	0.3 ± 0.8	0.463
Right side	0.1 ± 0.2	0.2 ± 0.8	0.473
Left side	0.1 ± 0.2	0.1 ± 0.3	0.896

SSL, sessile serrated lesion.

$P < 0.05$ was considered statistically significant.
